# Normative measurements of the frontal nerve by magnetic resonance imaging in an Australia cohort

**DOI:** 10.1007/s00276-025-03573-4

**Published:** 2025-01-29

**Authors:** Dinan Perera, Khizar Rana, Carmelo Caltabiano, Sandy Patel, Dinesh Selva

**Affiliations:** 1 Department of Ophthalmology & Visual Sciences, University of Adelaide, North Terrace, Adelaide, SA 5000 Australia; 2 South Australian Institute of Ophthalmology, Royal Adelaide Hospital, Port Road, Adelaide, SA 5000 Australia; 3 Adelaide Health and Medical Sciences Building, 4 North Terrace, Adelaide, 5000 Australia

**Keywords:** Frontal nerve (FN), Normative measurements, IgG4-related ophthalmic disease (IgG4-ROD), Nerve enlargement

## Abstract

**Purpose:**

To report the normative dimensions of the frontal nerve (FN) on fat-suppressed suppressed gadolinium (fs-gad) enhanced magnetic resonance imaging (MRI).

**Method:**

A retrospective cohort study of patients who underwent coronal fs-gad T1-weighted MRI. Orbits were excluded if there was unilateral or bilateral pathology of the FN or optic nerve sheath (ONS), incomplete MRI sequences, poor image quality or indiscernible FN on radiological assessment. The maximum diameter of the FN and ONS was measured.

**Results:**

The mean age of participants was 58 ± 16 years and 50% were females (n = 42). The mean normative measurements (mean ± standard deviation) on coronal T1-weighted imaging: optic nerve sheath, 5.08 ± 0.67mm. On coronal fs-gad T1-weighted imaging: frontal nerve, 0.74 ± 0.18mm. No significant differences were found between male or female participants in both the frontal nerve (p = 0.913) or optic nerve sheath (p = 0.646). There was no significant correlation between age and mean diameter of the frontal nerve (r = 0.14, p = 0.067) or optic nerve sheath (r = 0.075, p = 0.336). Additionally, no significant difference was identified between the mean diameter of the frontal nerve (p = 0.075) and optic nerve sheath (p = 0.120) across age groups. The mean frontal nerve to optic nerve sheath ratio was 0.15 ± 0.04.

**Conclusion:**

Normative dimensions of the FN may provide quantitative cut-offs that can aid the diagnosis of FN enlargement seen in instances such as IgG4-related ophthalmic disease (IgG4-ROD) and neoplastic perineural spread.

## Introduction

The frontal nerve (FN) is the largest branch of the ophthalmic division that continues from the trigeminal nerve. The FN enters the orbit through the superior orbital fissure and courses between the lacrimal nerve and the superior ophthalmic vein [1, 5, 8]. It travels superficially along the surface of levator palpebrae muscle, bifurcating into the supratrochlear and supraorbital nerve at variable lengths [1, 5, 8]. Both the skin of the scalp and forehead are innervated by these nerves [8].

IgG4-related ophthalmic disease (IgG4-ROD) is a lymphoproliferative condition that accounts for 11–43% of all orbital lesions [10]. Trigeminal perineural disease is a common orbital manifestation of this condition due to the infiltration of IgG4-positive plasmacytes in the epineurium of nerve fibres, causing nerve enlargement [16]. Whilst plasmacytes primarily infiltrate the infraorbital nerve, studies have shown that it also affects the FN [3, 6, 7, 9, 11, 17, 18, 20]. In a cohort study involving 115 IgG4-ROD patients with pre-operative orbital imaging, 15% (n = 17) of patients exhibited FN enlargement [9]. Other conditions with reports of FN enlargement include adult-onset xanthogranulomatosus [14], schwannoma [21] and lymphoma [9]. These cases illustrated unilateral enlargement, whereas bilateral FN enlargement was specific to IgG4-ROD [3, 7, 9, 17, 18, 20].

Traditionally, FN enlargement is defined when the diameter of the nerve is greater than the optic nerve sheath (ONS) diameter in the coronal section [9]. Currently, there is no literature reporting the normative values of the FN, which would allow for a more accurate assessment of enlargement. Therefore, the aim of this study is to present the normative dimensions of the FN in an Australian cohort on high resolution orbital MRI.

## Methods

### Subjects

A retrospective review of MRI scans was performed on a subject pool of 510 orbits from 255 patients between September 2021 to December 2023. These scans were performed at the Royal Adelaide Hospital and indicated for patients with suspected orbital pathologies. The study adhered to the principles of the Declaration of Helsinki and was approved by the Central Adelaide Local Health Network ethics committee. Inclusion criteria included patients who were ≥ 18 years of age and had both T1-weighted and fat suppressed (fs) contrast-enhanced with gadolinium (gad) T1-weighted high-field (3 Tesla; 3 T) MRI of the orbits in coronal planes. Exclusion criteria included patients with bilateral or unilateral orbital pathology that affected the optic nerve sheath (ONS) and/or the FN, incorrect imaging sequences, poor image quality and difficulty in identifying the boundaries of the nerve. Two independent reviewers were involved in collecting the measurements and verified by a neuro-radiology consultant.

### MRI examination

Patients were evaluated using a Magnetom 3T Skyra scanner (Siemens, Germany) with a conventional turbo spin-echo sequence (TR/TE, 500/15; field of view, 200 × 200mm; matrix, 512 × 512). All patients’ scans were standardised, with axial sections obtained parallel to the optic nerve, and coronal sections positioned perpendicular to the axial plane. Contrast-enhanced images were obtained following intravenous administration of standard weight-based dose of gadolinium. All measurements were performed on high-resolution picture archiving and communication system (PACS).

### Nerve analysis

**Fig. 1:**
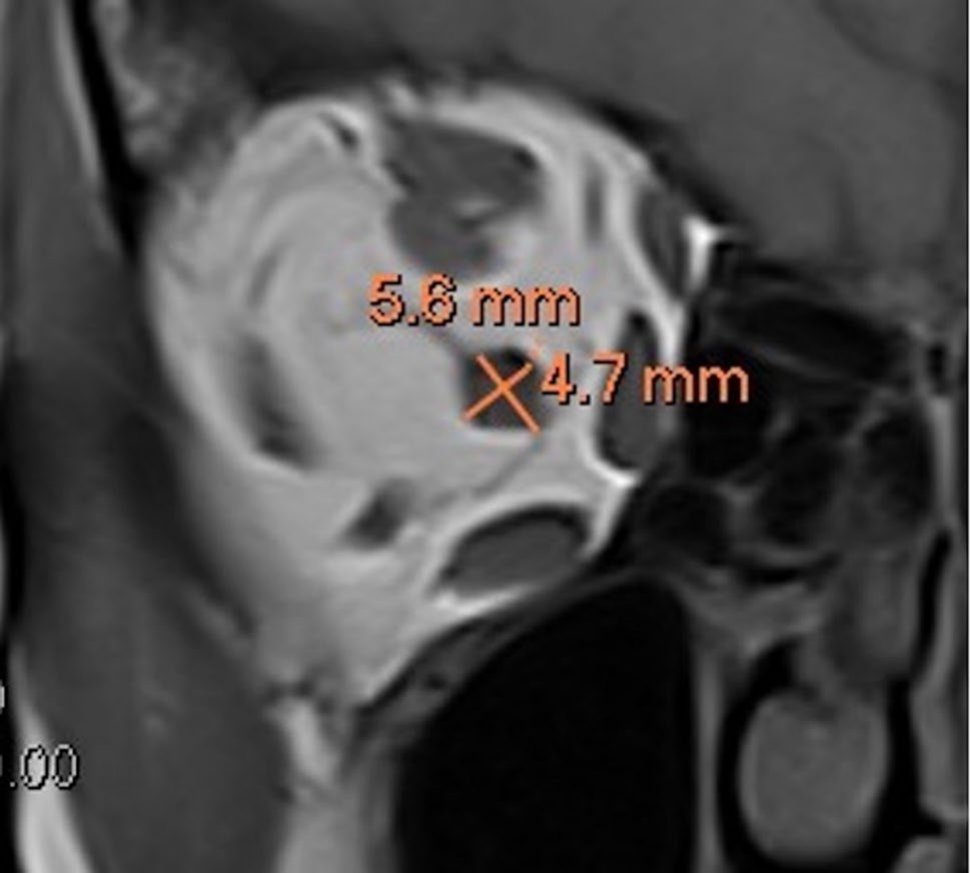
Coronal T1-weighted magnetic resonance imaging showing the short-axis dimension, measured perpendicular to the long-axis, of the optic nerve sheath

**Fig. 2:**
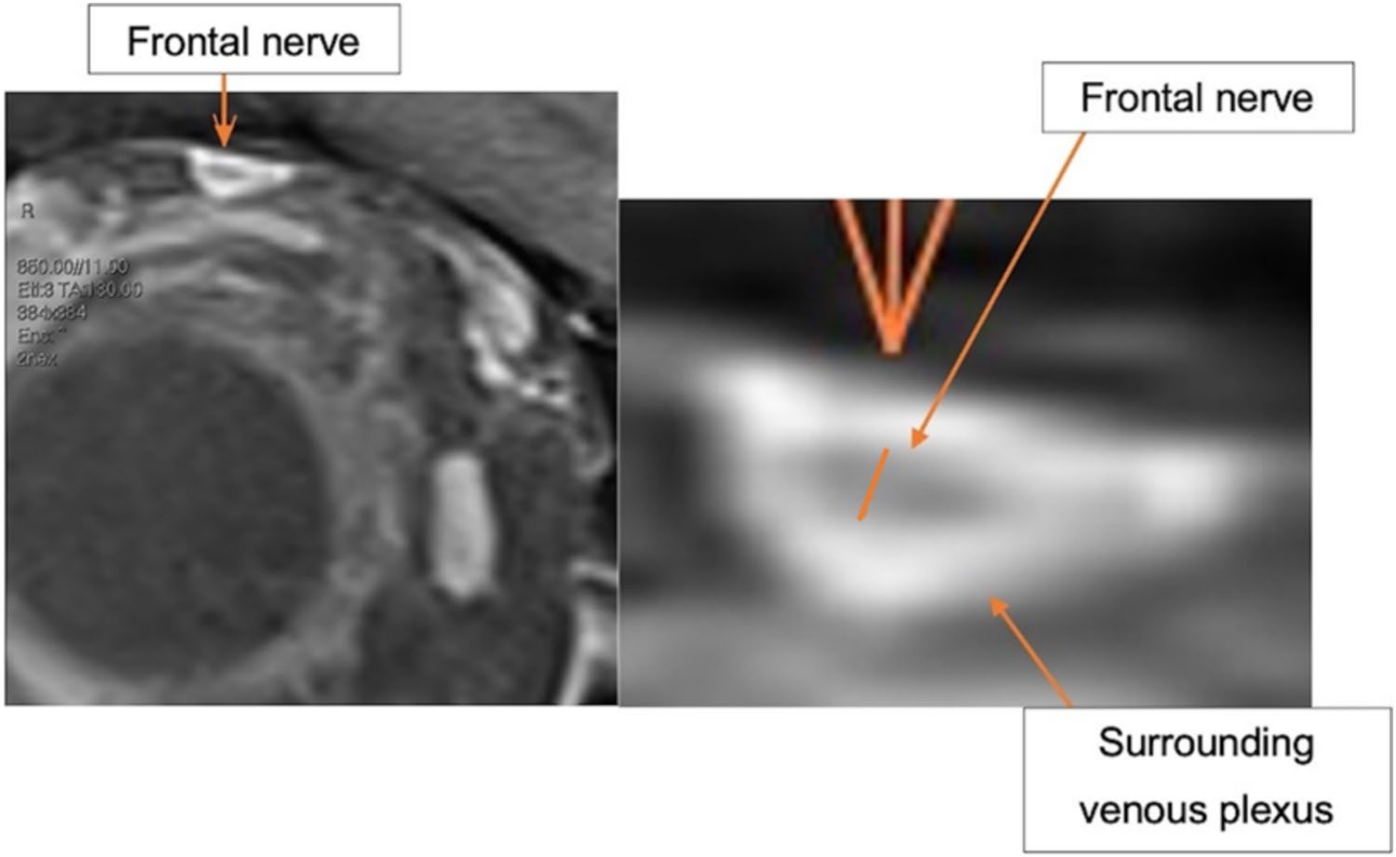
Coronal fat-suppressed contrast-enhanced T1-weighted magnetic resonance imaging illustrating measurements of the frontal nerve

The FN was identified using the coronal fs-gad T1-weighted MRI sequence, while the optic nerve sheath was identified using the coronal T1-weighted MRI sequence as its margins are more clearly delineated on this sequence [13, 15]. The FN was identified as the hypointense structure which ran supero-lateral to the superior ophthalmic vein. For both nerves, the slide in which the nerve was most apparent was selected and the maximum diameter of the nerve, perpendicular to the long axis, was then calculated (Figures 1 and 2). The surrounding enhancing venous plexus was excluded from the measurements. In cases where the nerve could not be confidently identified from surrounding structures, assistance from a neuro-radiologist was sought to clarify the FN boundaries.

### Statistical analysis

All statistical analyses were performed using IBM SPSS Statistics for MacOS, Version 27.0 (IBM Corp., Armonk, NY). The level of statistical significance was set at a *p* value of less than 0.05. Combined orbits are based on measurements taken from both the right and left orbit. The Shapiro–Wilk test for the right ONS showed evidence of normality (W = 0.99, p = 0.96), whereas the left ONS (W = 0.97, p = 0.02) and both left (W = 0.93, p =  < 0.01) and right (W = 0.95, p < 0.01) FN demonstrated evidence of non-normality. Therefore, the Mann–Whitney U test was used to evaluate the difference in FN measurements based on sex and laterality. Spearman’s coefficient was used to assess the correlation between age and the measurements of both FN and ONS. An independent samples Kruskal–Wallis test was used to compare the means of FN and ONS between different age groups. Intraclass correlation coefficient (ICC) was used to evaluate the interrater reliability of the two reviewers. Twenty scans were assessed by a second reviewer (CC) who was blinded to the results. The following ICC interpretation was used: poor (< 0.50), moderate (0.50–0.75), good (0.75–0.90) and excellent (> 0.90).

## Results

**Fig. 3:**
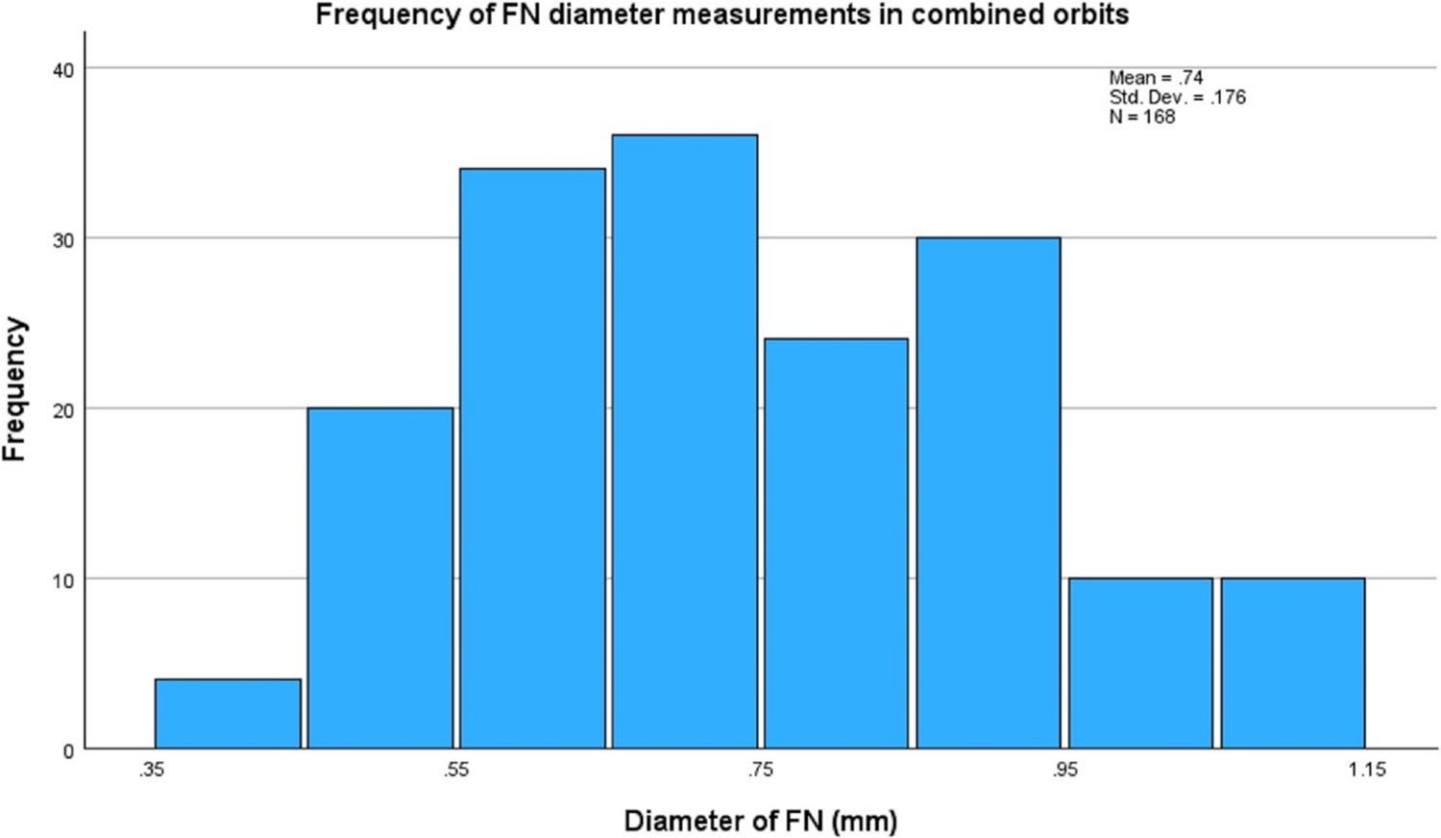
Histogram of frequency distribution of nerve diameters for the FN in combined orbits

**Fig. 4:**
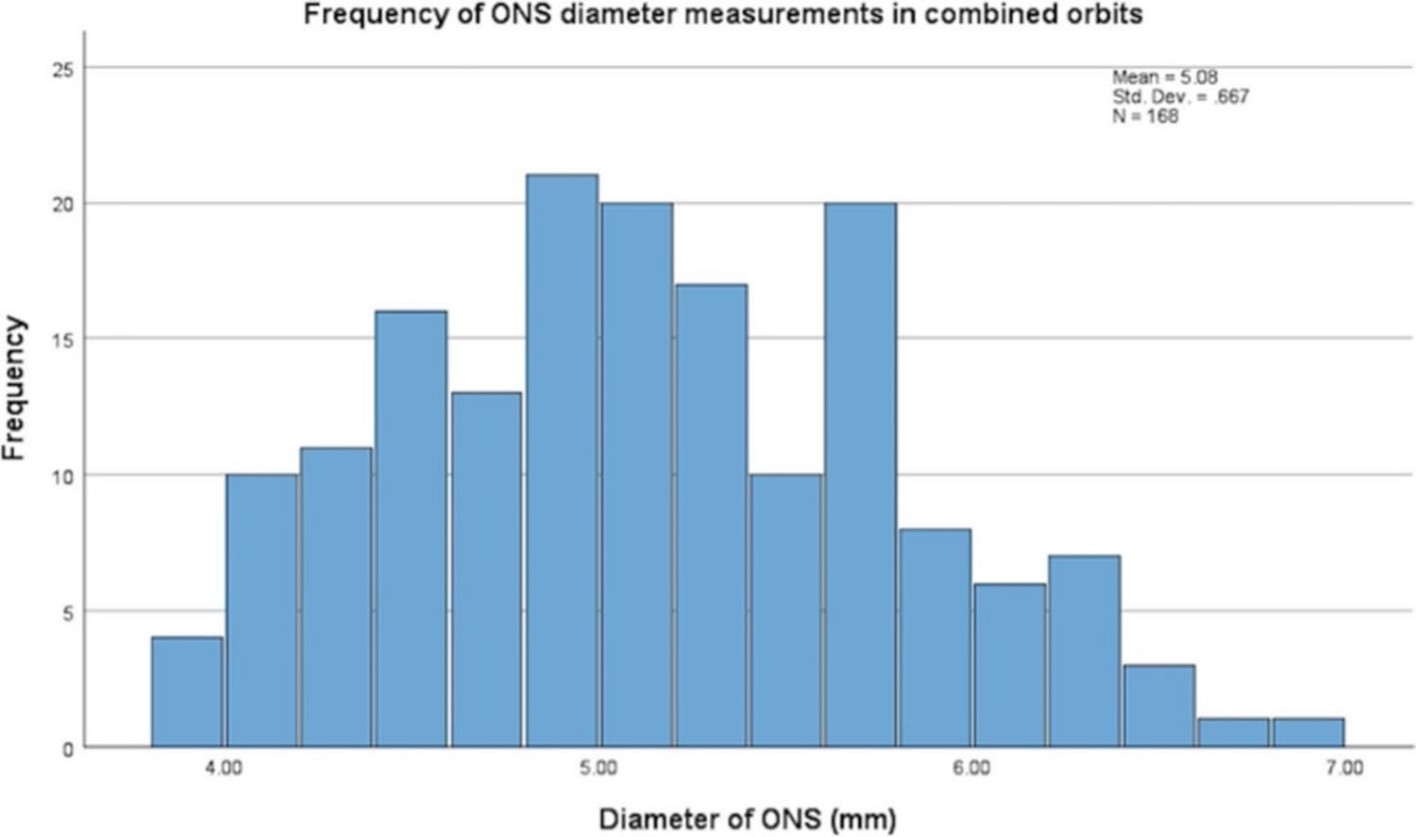
Histogram of frequency distribution of nerve diameters for the ONS in combined orbits

One hundred and sixty-eight orbits (84 left and 84 right) from 84 patients (42 male and 42 female) were included and 171 patients being excluded. The mean age of the participants was 58 ± 16.0 years (18–89 years). The mean diameter and standard deviation for the FN and ONS for all participants, including male and female, is included in Table 1. There was no statistically significant difference found in the FN and ONS diameters between males and females. The frequency distribution of nerve diameters for both the ION and ONS is displayed in Figure 3 and 4 below. The mean diameter of the right and left FN was 0.74 ± 0.18mm and 0.73 ± 0.18mm respectively. Mann–Whitney U test showed no statistical significance between measurements in the right and left orbit (p = 0.636) and laterality of the FN. Therefore, the combined orbit measurements were utilised for the remainder of statistical analysis, which is derived from both the right and left orbits.

**Table 1:** Normative measurements of the FN and ONS (mean ± SD) on magnetic resonance imaging, categorised by sex and age-groups. The comparison between sexes was conducted using the Mann–Whitney U test and the comparison between age-groups conducted using Kruskal–Wallis

Nerve	Total (n = 84)	Sex		*p* value	Age groups				*p* value
		Male (n = 42)	Female (n = 42)		18–39	40–59	60–79	80–99	
Frontal (mm)	0.74 ± 0.18	0.74 ± 0.17	0.74 ± 0.17	0.913	0.67 ± 0.15	0.73 ± 0.19	0.77 ± 0.17	0.72 ± 0.16	0.075
Optic nerve sheath (mm)	5.08 ± 0.67	5.10 ± 0.60	5.06 ± 0.73	0.646	4.78 ± 0.16	5.09 ± 0.86	5.19 ± 0.09	4.89 ± 0.95	0.120

**Fig. 5:**
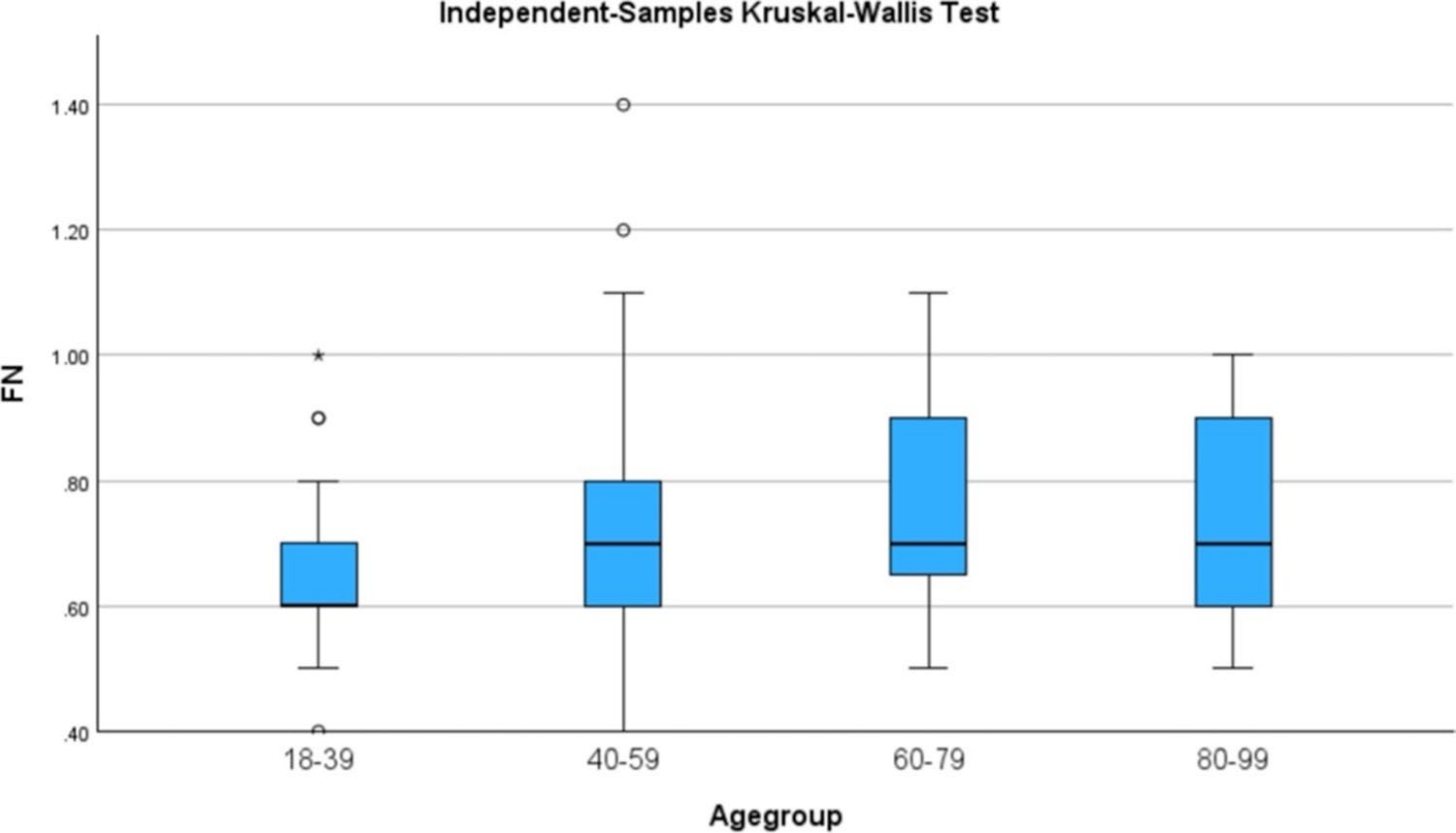
Comparison between diameter of the FN in combined orbits with differing age group, conducted using the Kruskal–Wallis test

The mean values of both FN and ONS across different age groups can be summarised in Table 1 and Figure 5. There was no significant correlation between age of participants and combined FN (r = 0.14, p = 0.067) or ONS (r = 0.075, p = 0.336) measurements through Spearman’s correlation coefficient. The Kruskal–Wallis test retained the null hypothesis, suggesting that the distribution of FN and ONS was not significantly altered across the age groups.

The mean diameter (± SD) of the FN to ONS ratio in combined orbits was 0.15 ± 0.04, with a range of 0.07–0.24 and an 95% confidence interval of 0.14–0.15. There was no statistically significant difference between the ONS to FN ratio in combined orbits and age groups (p = 0.449) or sex (p = 0.663).

The interrater reliability for average measures was good for both the ONS (ICC 0.868 for right and 0.861 for left) and the FN (ICC 0.841 for right and 0.895 for left).

## Discussion

Our study reports the normative coronal dimensions of the FN on fs-gad T1-weighted MRI, while also investigating its relationship with age, sex and ONS size. This study is the first to present normative FN measurements across any imaging modality. Additionally, we utilised high-resolution MRI scans in over 160 orbits. These findings may aid in defining a quantifiable definition for FN enlargement.

The upper ranges of the FN measurements, set at two standard deviations above the mean, could serve as a suitable criterion for defining the threshold of FN enlargement. Two standard deviations above the mean diameter were chosen to define abnormal enlargement [4, 13]. A diameter greater than 1.1mm in the coronal plane for the FN may be considered enlarged. Current definitions of enlargement require the FN’s diameter to be greater than that of the ONS, which is around 5mm. Our proposed normative data suggests that this criterion may under report the prevalence of FN enlargement.

One previous study measured the size of an enlarged FN, reporting dimensions of 20.6mm × 31.8mm [21], however no previous studies have reported the normative values. Therefore, a quantitative cut-off for FN enlargement has yet to be established. In our study, the FN and ONS measurements were taken at its maximum diameter as this can be used to help define abnormal enlargement [19]. The infraorbital nerve is the most common trigeminal branch lesion associated with IgG4-ROD [3, 18, 20], and its involvement is a prognostic indicator of IgG4-ROD and correlates with serum IgG4 levels and occurrence of multi-organ involvement [21]. Further studies may investigate if frontal nerve enlargement, defined using our normative data, may also serve as a marker of disease severity in IgG4-ROD.

Our study found no significant difference in FN diameter with respect to age group, sex, or laterality. Further studies across different centres are required to corroborate our findings. In addition, data on the ethnicity of the participants, which was not available in our study, would also be helpful to see if the diameters vary across different ethnic groups.

As current definitions define FN enlargement in relation to ONS size, this study provides the normative values for the FN:ONS ratio in both orbits. Given nerve size variability among individuals, the FN:ONS may be used as another quantitative indicator of FN enlargement. There was no significant difference in the FN:ONS between age groups or sex.

Coronal fs-gad T1-weighted MRI is the preferred imaging sequence for evaluating inflammatory and neoplastic conditions of the orbit. High-resolution volumetric T1-weighted images post-contrast enables for visualisation of trigeminal nerve branches. The frontal nerve is surrounded by a venous plexus which enhances, with the frontal nerve itself being relatively hypointense. Additionally, as the peripheral segments of the trigeminal nerve are surrounded by fat, employing fat-suppression sequences allow for clearer delineation of the FN [2]. Utilisation of high-resolution orbital MRI scans on 3T scanners allowed identification of the nerve in majority of cases (84/89 patients). However, five cases had unclear boundaries on two reviewers, including a neuroradiologist (SP) and therefore were excluded. MRI sequences with lower spatial resolution (e.g., 1.5 T) may see a higher proportion of cases where the boundaries of the frontal nerve may not be able to be distinguished.

Other causes of FN enlargement include adult-onset xanthogranuloma [14], lymphoma [9], schwannoma [21], idiopathic orbital inflammation, and neoplastic perineural spread from squamous cell carcinoma [12]. All of these cases had reported unilateral enlargement of the FN and are much less common than IgG4-ROD. The presence of bilateral trigeminal branch enlargement is specific for patients with IgG4-ROD [9, 17], with an approximate prevalence of 53% [9]. Therefore, the establishment of the normative diameter of the FN, along with the ability to assess bilateral FN enlargement on MRI, may help facilitate diagnosis of IgG4-ROD.

This study has limitations apparent in its design. The study is retrospective, based on patients from a single cohort which may not be applicable to other population groups.

Furthermore, this study did not comment on the shape of FN when measuring its diameter. In addition, frontal nerve and optic nerve sheath diameter may vary across its course and future studies could look at its dimensions at different points in its course.

In conclusion, we present the normative FN diameter in an Australian cohort using the fs-gad T1-weighted MRI. The data provides a cut-off of values that can be used to quantitively diagnose FN enlargement.

## Data Availability

No datasets were generated or analysed during the current study.
